# Supplementary material for: An Exploration of Environmentally Sustainable Practices Associated with Alternative Grazing Management System Use for Horses, Ponies, Donkeys and Mules in the UK
Source: Animals (Basel). 2022 Jan 8;12(2):151. doi: 10.3390/ani12020151 (PMC8772570; doi:10.3390/ani12020151)
Supplement: Supplementary file 1 [file animals-12-00151-s001.zip › Supp item S1.pdf]

Alternative equine care and grazing practices in the UK: Introduction

The University of Liverpool is working with the UK's equine charities, including the Horse Trust, Redwings, World Horse Welfare, Bransby, Blue Cross, the BHS and Donkey Sanctuary, to learn more about how horse, pony, mule and donkey owners use different grazing practices to manage their horses. We would particularly like to learn more about the alternative grazing practices used in the UK, for example track systems, rewilding projects, equicentral systems or other. Therefore, we are asking the equine community to share their experiences with these systems. We are interested in positive, negative and neutral thoughts and experiences.

Information you provide will be compiled into a report which will be made freely available to the equine community. This report will aim to share best practice and lessons learnt from those using alternative grazing practices to manage their horses. You are under no obligation to take part, but we are of course very grateful for any information you send.

This questionnaire is open to **all UK horse, pony, mule and donkey owners who use a grazing system they consider "alternative"** (e.g. a track, Equicentral or rewilding project).

Reading this information and completing the survey will be considered as consent to participate in this study. **You must be at least 18 years old to participate.** This project has been fully approved by the ethics committee at the University of Liverpool.
Thank you in advance for your participation.

Why am I being invited to take part and what will happen if I decide to participate?

You are being invited to take part because you are a UK horse owner who uses an "alternative" grazing system such as a track system, Equicentral or rewilding system.

If you decide to take part you will need to complete the following questionnaire, which will take around 15-20 minutes.

Participation is **entirely** voluntary and you do not have to take part in the study. You do not have to give a reason if you do not wish to take part.

You are free to withdraw at any time until you have selected the 'Finish' button on the final page of the questionnaire, after which it will not be possible to withdraw responses.

How do I answer the questions?

Please answer the questions by selecting the appropriate answer box. Some questions will allow you to select more than one answer. For answers where more detail is required we have provided a box for you to type your answer. We know that these systems are very important to some owners, so if you'd like to share more comments, thoughts, images or diagrams you are welcome to email tfurtado@liverpool.ac.uk

How will my data be used?

All data is strictly confidential, will be used for this specific project only, and will be available only to the investigators. Data will be aggregated and no individuals will be

identifiable from any published data. It is possible that we might use quotes or images in the report, to be shared with other horse owners (although we will not include your name, location etc).

The data you provide will be stored securely for 7 years, in line with the data protection requirements at the University of Liverpool and GDPR.

What if I am unhappy or there is a problem?

If you are unhappy, or there is a problem, please contact the researchers listed below and we will try to help. If you remain unhappy or have a complaint which you feel you cannot communicate directly to our researchers then you should contact the Research Ethics and Integrity Office on 0151 794 8290 (ethics@liv.ac.uk).

When contacting the Research Governance Officer, please provide details of the name or description of the study so that it can be identified, the researcher involved, and the details of the complaint you wish to make (study name: Alternative Grazing Systems in the UK; researcher name: Dr Tamzin Furtado)

Who can I contact for further details?

- **Dr Tamzin Furtado**

Institute of Infection and Global Health, University of Liverpool Leahurst Campus, Chester High Road, CH64 7TE.

Email: tfurtado@liverpool.ac.uk

Consent to participate

Please confirm that you have read and understood the above information, are over 18 years old and consent to participating in this study:

[tick box] I have read the above information and I consent to participating in this study. *Required*

Before we talk about the grazing system – a bit about you:

1. Age category 18-25, 26-34, 35-44, 45-54, 55-64, 65-74, 75-84
2. Years of involvement with horses: <1, 1-2, 2-5, 5-10, 10-20, 20+
3. Please let us know the first part ONLY of your post code, e.g. LL13
4. What is your relationship with the alternative system (by “alternative system” we mean the track, Equicentral, rewilding project, or other grazing system)?
    a. I run the alternative system myself and charge others to keep their animals here
    b. I run the alternative system for my own personal animals only
    c. I am a livery at an alternative system

d. Other (please explain)

Part 1: The physical environment of your set-up:

1. Roughly how much land is available to you for your grazing system? (acres)

2. How long has this system been in place?

3. Do the horses/ponies/mules/donkeys on this system have any access to grass? (if no, please provide a description of the surface used)

4. What type of fencing do you use? Please describe (if electric fencing, please specify whether you have the electric on or off)

5. If you use tracks, approximately how wide are those tracks? (m)

6. Do your animals have access to shelter? Please describe

7. Do you provide any enrichment on your track systems (e.g. “entertainment” or positive welfare items for horses/ponies/mules/donkeys such as treat balls, scratching posts, herb gardens, different surfaces etc) – if yes please describe

8. Please describe how your grazing system **looks** - for example do you use tracks around fields in a square? Are there different surfaces in different places? Do you have hardstanding areas or “loafing” areas? Note: you can include photos or diagrams if you wish; please indicate whether you are happy for them to be used in the report and any associated media, e.g. social media. By consenting you therefore confirm you have all necessary permissions for the photographs or diagrams to be shared

Part 2: how you manage your set up

9. How many horses do you keep on the alternative system?

10. Do your horses receive additional forage, such as hay or haylage? Yes all year/ yes part of the year/ no

    a. If yes, what forages do you use?
    b. Is this supplementary forage fed ad-lib?
    c. How do you feed these forages (e.g. on the ground, hay feeder, trickle nets etc)

11. Do you monitor the weight of the horses/donkeys/mules/ponies on your system? If so, how do you measure their weight, and how regularly?
12. If yes, has the weight or body condition of your animals increased or decreased since implementing the grazing system?

13. Do you aim to use the system specifically as a form of weight management (reducing weight)? If so, please tell us your experience of the system for weight management.

14. Do you use the system to manage other health conditions? If so, what?

15. Please describe how you **manage** the animals on your grazing system - for example, do you change things so that horses access different areas at different times? Do you still use the set-up in winter? Are any of the horses on your system stabled at any times? Do you use other animals such as sheep to manage grass?

Part 3: General questions:

16. What were the reasons that you set up this alternative system initially?

17. What are the best things about your alternative grazing system?

18. Have you encountered any difficulties with the system, or do you have any concerns about their use?

19. If you had to give advice to someone thinking of using an alternative system like yours, what would you tell them?

If you have any extra comments, please feel free to share them here.

Thank you for your time taking part and sharing your experiences. Please feel free to share this survey widely. We will be collecting responses from participants for a period of one month: after this time, we will close the survey, anonymise all the data, and compile a report of the experiences of horse owners and managers of these systems.

If you'd like a copy of the report once it is ready, please leave your email here. Your email address will be used only to send a copy of the survey, and will not be linked to the answers you've given here:
