# Supplementary material for: An Exploration of Environmentally Sustainable Practices Associated with Alternative Grazing Management System Use for Horses, Ponies, Donkeys and Mules in the UK
Source: Animals (Basel). 2022 Jan 8;12(2):151. doi: 10.3390/ani12020151 (PMC8772570; doi:10.3390/ani12020151)
Supplement: Supplementary file 1 [file animals-12-00151-s001.zip › Supp item S2.pdf]

Supplementary item S2

Description of Equicentral, track and rewilding use by respondents

Type of system	Description of system	Positive aspects	Limitations	Usage:
Track system	Track systems involve the horses living on a track, usually around the perimeter of a field or several fields. Ideally, this track has low levels of grass or no grass at all, and resources such as hay, water, shelter, and enrichment are distributed around the track area. This purportedly encourages horses to move more than they would in a standard square field environment. The central area is usually left to grow as standing hay. This crop is sometimes kept as "standing hay" for winter use, or sometimes made into hay.<br>Proponents of tracks recommend that some areas of the track are wider (e.g. corner areas, areas of shelter) to ensure that there is adequate space for horses to move away from one another.<br>Many respondents removed the track in winter, replacing it in spring, due to concerns about mud in winter. Track users who did not do this were generally able to use surfaced tracks rather than grass or dirt.	• Economical use of land<br>• Provides a low-grass environment with maximisied movement (considered ideal for horses with equine metabolic syndrome, for example).<br>• Herd living, with flexibility (for example, horses with additional forage needs can easily be kept on the interior paddock, while others remain on the track)<br>• Enrichment was easily provided: numerous examples were provided, ranging from sand pits to herb gardens,	• Workload: tracks required a relatively high workload in terms of needing to poo-pick a wide area on a daily basis, refill hay feeders, etc.<br>• Careful nutrition: some horses were limited to predominantly eating soaked hay; careful attention to nutrition was necessary to ensure that the horses' nutritional needs were met.<br>• The need to be in control of the land: Setting up a track system was difficult for those who rented land, because land owners purportedly	Most common usage:<br>• 3-5 horses<br>• 2-3 acres of land<br>• 66% of respondents who used tracks were in control of the land (e.g. owned/rented)<br>• 8% had entirely grass-free tracks<br>• Most commonly used to manage laminitis (29.5% of respondents), arthritis (17.4%) and EMS (16.8%)<br>• On average, track users had their tracks at around 4m in width, but this varied even within one single system.<br>• 65% of track users fed supplementary forage all year round, with 17%

	Track system users preferred their horses to eat high fibre, low energy forage, and hence removed grass as much as possible (either by using surfaces such as gravel, by strip grazing, or through co-grazers such as sheep) and instead feeding low energy forage (e.g. soaked hay), which could be placed at different points on the track.	paddling pools, logs, steps, hills, puzzle feeders, hedgerows, scratching posts, flavoured waters, and many more.<br>• Centre area is usually rested, and hence grows mature, biodiverse flora which was considered to increase wildlife	found it hard to understand the reason for the bare ground caused by the track.<br>• Cost: the costs of fencing, feeding hay, and surfacing meant that the investment in tracks could be high, although it was easy to set up a simple track to begin with.<br>• Environment/soil management: there was some concern from respondents that the track area is essentially sacrificed, with heavy footfall and close grazing. This could lead to poor soil quality and increase in weeds. However, the central area tended to be little grazed and therefore encourage biodiversity and soil health, so this	feeding part of the year.

			seemed to be down to individual opinion and dependent on the way the track was set up.<br>• Some track users created complicated shapes such as spirals and zig zags to encourage movement. This was a concern for behaviourist experts, who suggested that horses might find complex set-ups such as spirals to be stressful.<br>• Because of the risks to horses from close confinement, it is important to ensure that tracks are not too narrow; however narrower tracks obviously mean even more grass, which could be problematic for those using tracks to manage weight	

			• Mud – many track users removed tracks in winter	
Equicentral	Equicentral is an idea pioneered by Stuart and Jayne Myers (read more at www.equiculture.net), which aims to balance the need to look after the land and support soil health, in order to better cater for the behavioural and health needs of the horses. It is similar to modern farming methods of "mob grazing" which let animals on pasture for short amounts of time, allowing the pasture and soil ample time for regrowth.<br>The system works by dividing the land into interlinked paddocks, which are rested frequently to ensure optimum grass and soil health. The heart of the Equicentral is a "loafing area"; an enclosed area which is usually surfaced, where the horses can rest, shelter, interact and eat hay. Some Equicentral supporters open up existing stable yards for this purpose, while others create surfaced areas around a field shelter. The surfaced area then acts as a "holding area", where horses can be kept off the grass for a period of time each day (or as the owner wishes). Gates are then opened at time periods (for example, for a few	• Looking after the land and environment: the foundation of Equicentral is about encouraging biodiversity and soil health. Grass is never over-grazed or allowed to become "horse sick". Proponents say that they see more wild plants and wildlife around their land as a result.<br>• Herd living, but with flexibility: proponents suggest that it is very easy to be flexible with different types of horses, while still maintaining herd life. For example, if one horse is getting too fat, it can easily be kept in the loafing area while	• Workload: Some owners suggested that Equicentral required a relatively high level of intervention; e.g. to be available at least twice daily to open/close paddocks, muck out the loafing area, and restock hay and enrichment. However, droppings removal was not as frequent as other systems<br>• The need to be in control of the land: several respondents suggested that they had difficulties because they shared their land with someone who had different ideas about equine management.<br>• Weight management: some	Most common usage:<br>• 3-5 horses<br>• 2-3 acres of land<br>• 77 of respondents who used tracks were in control of the land (e.g. owned/rented)<br>• Most commonly used to manage laminitis (28.5%), arthritis (16.3%), EMS (13.5%)

	hours each day) to allow the horses to graze in well-rested paddocks. Proponents of this system place great importance on maintaining the soil and grass health, by resting the fields appropriately; they suggest that horses should not be turned out on grass less than 5cm in height. Therefore, paddocks are usually split into several (some users reported having up to 20 different areas). Sometimes these were arranged as “pizza slices” from the yard, while others had tracks which allowed horses to access different areas, or simply used electric fencing to move the area which the horses could access.	the others have access to the grass.<br>• Lack of mud: if maintained in the way Equicentral suggests, the poaching of land is limited because horses do not spend time hanging around in gateways; they go out into the fields, eat, then come back to the non-grass loafing area.	were concerned that horses would binge-eat when allowed on the grass; some respondents felt that the system was difficult to use with overweight horses. However, others described that their horses were calmer and less stressed generally on this system because of its careful set-up, and because they ate plenty of hay in the loafing area, did not seem to binge eat when they were turned out. Also, the use of the loafing area meant that it was very easy for some respondents to keep horses on non-grass turnout, if needed.<br>• Cost: the emphasis on non-grass areas inevitably meant that many	

			respondents needed to invest in mud control mats, shingle, pea gravel, limestone, or other means of hand-standing, unless they were lucky enough to have an existing yard to use. This, and the cost of extra fencing and hay were quoted by many as limitations of the system, although most commented that these costs were outweighed by its benefits.	
Rewilding or conservation approach	"Rewilding" is part of a philosophy which considers that human management of land often takes away more than it gives back to the environment, but that nature has its own holistic ecosystems whereby soil, plant life, insect life, and wildlife can flourish without human intervention. Because horses survive very well in "wild" conditions (feral, in the UK), rewilders suggest that horses can be kept as part of this holistic system.	• Enjoyment of the environment: Rewilding horse care offered the chance for owners to engage with the entire ecosystem of environmental wellbeing, and many owners mentioned their enjoyment of watching unusual	• The need for ample land: rewilding will only work in areas where the land is not overburdened by horses; many yards could simply not manage this system based on the horse:land ratio.<br>• Weight management:	Most common usage:<br>• 3-5 horses<br>• 7-10 acres of land<br>• 75% were in control of their own land (e.g. owned/rented)<br>• Most commonly used to manage laminitis (31.5%), arthritis (16.4%), EMS (13.7%)

	Rewilding involves letting the land recover from human intervention, and supporting the growth of local flora and fauna. Over time, the diversity of wildlife and plants will increase dramatically. Traditionally, horse keeping involves intensive grazing of monocultures, which is detrimental to soil health and local ecosystems. Rewilding projects therefore reverse this trend by allowing the land to return to a more “natural” state with greater diversity.<br>Participants in this study showed two schools of thought around how to combine rewilding with horse care. Full rewilders generally had a large amount of acreage per horse and discussed creating entire ecosystems which went far further than just providing a place for horses to live; instead the horses were an integral part of a wider ecosystem involving the land, wildlife, insect life and plants. Partial rewilders often had less space per horse (though still more space than for other systems). Because intensive horse footfall damages the land, people therefore incorporated *aspects* of rewilding into their horse care. For example, they kept the horses off land all summer and allowed the land to rewild itself in the meantime, or	flora and fauna thrive in the environments they had allowed to grow.<br>• A natural life for horses: While all the systems in this project suggested that they have some elements of being “natural”, the rewilders surely win the prize for creating an environment as close as possible to feral/wild life for a horse. Horses were often completely unrestricted, and could choose whether or not to seek resources such as shelter, additional hay and so forth.<br>• Horse health: Users of these systems often had native ponies (e.g. those mentioned included Shetlands, Exmoor,	because of being relatively unrestricted, horses could put on excess weight on this system. Several respondents suggested that their native breeds self-regulated, but this is very dependent on the individuals and environment. Other horse owners needed to keep their horses off the wild areas during spring and summer, in order to manage their weight.<br>• Monitoring for dangerous plants or areas: it is important to avoid the proliferation of dangerous, poisonous or invasive plants and watch out for potentially hazardous areas.<br>• Mud: depending on the land and	

	alternatively they had sections of their land which were dedicated to rewilding, and the horses were allowed only infrequent access to these areas so as not to damage them.<br>For all respondents, the aim of rewilding was to balance care of the land with the care of their horses. Generally, horses were allowed access to large, open spaces (sometimes 15-20 acres or more) with diverse plants, rough grazing, and relatively little intervention compared to other systems. If needed, some respondents also utilised hard standing or partial stabling to minimise impact on the land.<br>To support the land, rewilders often also utilised co-grazers such as pigs and cows, who could help "plough" the land, and whose droppings could help fertilise. Some respondents did not poo pick their horses' droppings, but this was dependent on the amount of space. Aside from removing poisonous plants, most rewilders allowed the land to care for itself – however, some preferred to remove the more pervasive, acidic-loving plants such as dock leaves and buttercups.	New Forest and Icelandic ponies), who were considered prone to laminitis and weight gain. However, most rewilding users suggested that their ponies were thriving on the diverse forage provided by this system, and losing weight in winter as they would if they were living in the wild. Of course, this would depend very much on the individual ponies and the land itself, so monitoring health was also important.<br>• A comparatively low workload: because rewilding systems rely on lack of human intervention, in an ideal world the land and horses could co-exist without too much effort for the owner	management, mud could be an issue; many relied on areas of hard standing to help manage this.	

		– for example, some owners did not have to poo-pick, manage fencing to any great extent, harrow or roll land etc. This only worked with larger systems; when less acreage was available, owners still had to conduct some pasture management such as poo picking, confining horses to hard standing and feeding hay, etc.		
